# State-Referenced Truncated SVD for Dynamic Microwave Monitoring of Intracranial Hemorrhage

**DOI:** 10.3390/bios16050285

**Published:** 2026-05-14

**Authors:** Zekun Zhang, Heng Liu, Ruide Li, Huiyuan Zhu, Fan Li, Shujun Ni, Aojun Liu, Yao Zhai

**Affiliations:** 1 School of Information and Electronics, Beijing Institute of Technology, Beijing 100081, China; 2 School of Cyberspace Science and Technology, Beijing Institute of Technology, Beijing 100081, China; 3 Yangtze Delta Region Academy of Beijing Institute of Technology, Jiaxing 314019, China; 4 Department of Nursing, College of Medicine, Jiaxing University, Jiaxing 314001, China; 5 Provincial Key Laboratory of Multimodal Perceiving and Intelligent Systems, Jiaxing University, Jiaxing 314001, China

**Keywords:** Born approximation, dynamic monitoring, intracranial hemorrhage, microwave imaging, state-referenced imaging, truncated singular-value decomposition

## Abstract

Microwave imaging is a promising non-ionizing technique for bedside follow-up of intracranial hemorrhage, but dynamic monitoring remains challenging under limited multistatic sampling because weak inter-frame changes can be obscured by measurement variability, model mismatch, and the high cost of frame-by-frame nonlinear inversion. To address this problem, this paper proposes a state-referenced truncated singular-value decomposition (SR-TSVD) framework for dynamic microwave monitoring of hemorrhagic evolution. The method maintains an internal gate state and reconstructs only the state-referenced increment at each monitoring instant. A row-whitened TSVD inversion is introduced to reduce channel dominance effects and improve robustness to route-dependent imbalance, while a residual-driven gate-refresh mechanism updates the internal state only when the current linearization background becomes insufficiently accurate. The proposed method was validated through two-dimensional numerical experiments and hardware phantom measurements. The numerical study examined different lesion evolution scenarios and analyzed the effects of antenna count, frequency diversity, and measurement noise. The hardware study showed that the method preserves the main dynamic evolution in a real measurement system and remains more stable than baseline linear methods under sparse array conditions. These results indicate that SR-TSVD provides an effective and computationally practical framework for repeated bedside microwave monitoring of intracranial hemorrhage.

## 1. Introduction

Intracranial hemorrhage is a time-critical neurological condition, and its clinical management depends not only on initial detection but also on repeated reassessment during the acute stage. At present, computed tomography (CT) and magnetic resonance imaging (MRI) remain the main tools for diagnosis and follow-up [1]. However, repeated bedside reassessment still motivates the development of complementary monitoring techniques that are easier to deploy [2]. Microwave imaging has, therefore, received sustained attention because it is non-ionizing, potentially portable, and directly sensitive to tissue-dependent electromagnetic properties [3]. Its biomedical basis can be found in early studies on microwave interrogation and diffraction tomography [4,5].

Microwave brain imaging relies on the contrast of complex dielectric properties among biological tissues. Extensive measurements over broad frequency ranges have shown that dielectric differences among tissues are large enough to support microwave sensing [6]. In the head, this contrast is embedded in a layered and heterogeneous environment that includes the skull, cerebrospinal fluid, and brain parenchyma [7]. Realistic head phantoms and anatomical models are, therefore, important for evaluating reconstruction methods under physically relevant conditions [8]. In hemorrhagic cases, the dielectric contrast between blood-related inclusions and surrounding brain tissue provides the basic sensitivity required for microwave detection and follow-up, while the same anatomical complexity makes stable reconstruction much more difficult [7,8].

Based on this physical foundation, many microwave head-imaging systems have been reported for stroke and traumatic brain injury applications. Early multistatic studies demonstrated the feasibility of microwave head imaging for stroke-related detection [9]. Later prototype work further explored practical head-imaging hardware for intracranial hemorrhage sensing [10]. Feasibility studies and early clinical or quasi-clinical investigations also showed that microwave systems can provide useful sensitivity to intracranial abnormalities in realistic settings [11,12,13]. More recent developments include three-dimensional prototype systems and portable platforms oriented toward stroke imaging or monitoring [14,15]. These studies show that microwave brain imaging has moved beyond basic feasibility studies and now faces more practical questions related to reconstruction stability, sparse-aperture design, and repeated monitoring.

Recent reviews have discussed these issues more clearly. Microwave brain imaging is now often assessed in terms of the balance among physical fidelity, computational efficiency, and system compatibility [16,17]. Related reviews on near-field sensing and microwave focusing methods also show that algorithm design must be considered together with device complexity, sampling density, and deployment constraints [18,19]. In this context, existing reconstruction methods may be broadly grouped into two classes.

One class emphasizes model fidelity through iterative inverse-scattering formulations. Representative examples include the Born iterative method (BIM) and the distorted Born iterative method (DBIM), contrast source inversion (CSI), and finite-element-based inversion schemes [20,21,22]. These methods have also been adapted to brain-imaging problems through Born-type iterations, distorted Born formulations, and experimentally validated microwave tomography pipelines [23,24]. More recent work has introduced clustering or multi-stage update strategies to improve stroke-oriented reconstruction and classification [25,26]. The main advantage of this class is its stronger treatment of multiple scattering and background updating. Its main limitation is the substantial computational cost.

The other class emphasizes lower complexity and faster imaging. Examples include frequency-based multistatic imaging, rapid migration-type methods [27], truncated singular-value decomposition (TSVD)-based linear inversions, and differential imaging strategies designed for repeated follow-up [28,29,30]. Comparative studies have shown that such methods can be attractive for compact systems and time-constrained applications, even though they usually rely on weaker scattering assumptions and are more sensitive to sparse sampling and measurement variability [31]. This difference becomes especially important in dynamic monitoring, where the quantity of interest is often not a large lesion reconstructed independently at every instant, but a relatively small change between successive frames.

Under sparse multistatic sampling, such weak inter-frame changes can be obscured by switching-dependent variability, coupling changes, imperfect calibration, and cumulative modeling mismatch. These factors are closely related to practical monitoring-oriented architectures, which often use limited antenna counts, sequential switching, and compact measurement subsystems in order to reduce hardware complexity and acquisition burden [32,33]. Multiport hardware with real-time capability has improved data acquisition efficiency, but it does not remove the sensitivity of sparse systems to route-dependent variability [34]. High-fidelity numerical studies have also shown that monitoring performance is strongly affected by sampling sparsity and system nonidealities [35]. In practice, this may appear as weak increments being masked, apparent location drift across frames, or fluctuation-induced artifacts that do not correspond to true lesion evolution. A direct adjacent-frame differencing strategy may, therefore, amplify instability, whereas a fully nonlinear iterative reconstruction at every frame may exceed the computational budget required for practical follow-up.

These points suggest the need for a reconstruction strategy that is explicitly matched to the monitoring task. Instead of reconstructing each frame independently or relying only on direct adjacent-frame subtraction, the proposed method maintains an internal reference state and updates only the incremental change relative to that state. For this reason, this paper proposes a state-referenced truncated singular value decomposition framework, termed SR-TSVD, for dynamic microwave monitoring of intracranial hemorrhage. At each monitoring instant, the new measurement is compared with the multistatic response estimated from the current gate state. The resulting differential inverse problem is solved by a row-whitened TSVD scheme, while a residual-driven refresh mechanism updates the gate state only when the current reference is no longer sufficiently accurate. This formulation is intended to retain the low cost of linear inversion on most frames while reducing the instability associated with direct frame differencing and avoiding frame-by-frame nonlinear re-solving.

The contributions of this paper are summarized as follows:

(1) A state-referenced dynamic reconstruction framework is established for sparse-array microwave monitoring, in which an internal gate state defines the current reference background and forms frame-wise state-referenced differential data.
(2) A row-whitened TSVD inversion and residual-driven gate-refresh strategy is introduced to improve temporal stability, reduce channel-dominance effects, and preserve computational efficiency under repeated measurements.
(3) The proposed method is validated through controlled numerical experiments and hardware phantom measurements. The numerical study examines dynamic monitoring performance together with the effects of antenna count, frequency diversity, and noise, while the hardware study evaluates consistency in a real measurement system and clarifies the practical role of SR-TSVD in sparse-array monitoring.

The remainder of this paper is organized as follows: Section 2 presents the state-referenced formulation, the row-whitened TSVD inversion, and the gate-refresh mechanism. Section 3 reports the numerical validation under controlled dynamic scenarios. Section 4 describes the hardware phantom experiments and the corresponding quantitative evaluation in the real measurement system. Section 5 discusses the practical interpretation, limitations, and future directions of the proposed framework.

## 2. Methods

This section presents the proposed state-referenced truncated singular value decomposition (SR-TSVD) framework for dynamic intracranial hemorrhage monitoring. SR-TSVD maintains a gate state indexed by *s* and reconstructs small gate-referenced increments at each frame *t*, which denotes one complete multistatic acquisition over all selected transmit–receive pairs and operating frequencies.

### 2.1. State-Referenced Differential Model and Gate Linearization

Under the $e^{-j\omega t}$ convention, the complex relative permittivity is written as

(1) $$\varepsilon_{r}(r,f)=\varepsilon_{r}^{\prime }(r,f)-\frac{j\sigma (r,f)}{\omega \varepsilon_{0}},\;\;\omega =2\pi f,$$

where $\varepsilon_{r}^{\prime }$ is the real relative permittivity, σ is the conductivity, and $\varepsilon_{0}$ is the free-space permittivity.

Let ${\left\{f_{k}\right\}}_{k=1}^{F}$ denote the set of operational frequencies used in data acquisition and inversion, and $S^{\left(t\right)}(f_{k})\in {\mathbb{C}}^{N\times N}$ denote the measured multistatic scattering matrix for an *N*-antenna array at frame *t* and frequency $f_{k}$, with only its off-diagonal channels retained for reconstruction. Accordingly,

(2) O={(i,j)∣i≠j},  M=|O|=N(N−1),

and let ${\mathrm{vec}}_{O}(\cdot )$ denote the vectorization operator that stacks the entries ${\left\{S_{ij}\right\}}_{(i,j)\in O}$ into an *M* × 1 vector.

Using the baseline background $\varepsilon_{\mathrm{bg},0}(r,f)$, the contrast is defined as

(3) $$\chi (r,f)=\frac{\varepsilon_{r}(r,f)-\varepsilon_{\mathrm{bg},0}(r,f)}{\varepsilon_{\mathrm{bg},0}(r,f)}.$$

At gate index *s*, the gate-referenced contrast increment at frame *t* is

(4) $$\Delta \chi^{\left(t|s\right)}(r,f)=\chi^{\left(t\right)}(r,f)-\chi^{\left(s\right)}(r,f),$$

where $\chi^{\left(s\right)}$ stores the current contrast estimate, and the corresponding gate background becomes

(5) $$\varepsilon_{\mathrm{bg},s}(r,f)=\varepsilon_{\mathrm{bg},0}(r,f)(1+\chi^{\left(s\right)}(r,f)).$$

A Method-of-Moments (MoM) solver computes the total fields $\left\{E_{i}^{\left(s\right)}(r,f_{k})\right\}$ and the corresponding multistatic response $S_{\mathrm{est}}^{\left(s\right)}(f_{k})$ under the current gate background. The state-referenced differential matrix is

(6) $$\Delta S^{\left(t|s\right)}(f_{k})=S^{\left(t\right)}(f_{k})-S_{\mathrm{est}}^{\left(s\right)}(f_{k}),$$

and the corresponding vectorized differential data are expressed as

(7) $$\Delta s^{\left(t|s\right)}(f_{k})={\mathrm{vec}}_{O}\;\left(\Delta S^{\left(t|s\right)}(f_{k})\right)\in {\mathbb{C}}^{M}.$$

Stacking all *F* frequencies yields

(8) $$\Delta s^{\left(t|s\right)}=\left[\begin{array}{c} \Delta s^{\left(t|s\right)}(f_{1})\\ \Delta s^{\left(t|s\right)}(f_{2})\\ \vdots \\ \Delta s^{\left(t|s\right)}(f_{F}) \end{array}\right]\in {\mathbb{C}}^{FM}.$$

Within one gate cycle, $\Delta \chi^{\left(t|s\right)}$ is assumed to remain sufficiently small so that a first-order distorted Born approximation about the current gate background remains valid. Under this assumption, the gate-referenced perturbation for each (i,j)∈O at frequency $f_{k}$ is written as

(9) $$\Delta s_{ij}^{\left(t|s\right)}(f_{k})\approx j\omega_{k}\varepsilon_{0}\int_{\Omega }\Delta \varepsilon_{r}^{\left(t|s\right)}(r,f_{k})E_{i}^{\left(s\right)}(r,f_{k})E_{j,\mathrm{adj}}^{\left(s\right)}(r,f_{k})dr,$$

where $\Delta \varepsilon_{r}^{\left(t|s\right)}(r,f_{k})=\varepsilon_{\mathrm{bg},0}(r,f_{k})\Delta \chi^{\left(t|s\right)}(r,f_{k})$, $\omega_{k}$ denotes the angular frequency associated with the *k*-th operating frequency, and $E_{j,\mathrm{adj}}^{\left(s\right)}(r,f_{k})$ denotes the adjoint receive field associated with channel *j* under the current gate state. In reciprocal media, the adjoint receive field is obtained by solving the same forward problem with antenna *j* used as the source. The linearized kernel is, therefore, constructed numerically under the current gate background, without using a closed-form homogeneous-background Green’s function.

When the operating frequency band is relatively narrow, the gate-referenced contrast increment is further approximated as weakly frequency dependent:

(10) $$\Delta \chi^{\left(t|s\right)}(r,f_{k})\approx \Delta \chi^{\left(t|s\right)}(r).$$

After discretizing the region of interest Ω into *P* pixels centered at ${\left\{r_{p}\right\}}_{p=1}^{P}$, with $\Delta A_{\mathrm{eff}}$ denoting the effective pixel area, the discrete contrast vector becomes

(11) $$\Delta \chi^{\left(t|s\right)}={\left[\Delta \chi^{\left(t|s\right)}(r_{1}),\ldots ,\Delta \chi^{\left(t|s\right)}(r_{P})\right]}^{T}\in {\mathbb{C}}^{P}.$$

The stacked gate-linearized system then takes the form

(12) $$\Delta s^{\left(t|s\right)}\approx A^{\left(s\right)}\Delta \chi^{\left(t|s\right)}+e^{\left(t|s\right)},\;\;A^{\left(s\right)}\in {\mathbb{C}}^{(FM)\times P},$$

where $e^{\left(t|s\right)}$ collects higher-order scattering truncation error, forward-model mismatch, and measurement noise. The corresponding discrete coefficient is

(13) $$A_{(i,j,k),p}^{\left(s\right)}=j\omega_{k}\varepsilon_{0}\Delta A_{\mathrm{eff}}\varepsilon_{\mathrm{bg},0}(r_{p},f_{k})E_{i}^{\left(s\right)}(r_{p},f_{k})E_{j,\mathrm{adj}}^{\left(s\right)}(r_{p},f_{k}),$$

where (*i*, *j*, *k*) indexes channel (i,j)∈O at frequency $f_{k}$. Within a gate cycle, $A^{\left(s\right)}$ remains fixed and is rebuilt only when the gate is refreshed.

### 2.2. Row-Whitened TSVD Inversion

The row magnitudes of $A^{\left(s\right)}$ may vary substantially across channels and frequencies, so a small number of strong rows can dominate the inversion. To reduce this imbalance, a diagonal row-rescaling matrix is introduced.

Let $a_{m}^{T}$ be the *m*-th row of $A^{\left(s\right)}$ and define

(14) $$\mu_{m}=\frac{1}{P}\sum_{p=1}^{P}|A_{m,p}^{\left(s\right)}|,\;\;w_{m}=\frac{1}{\mu_{m}+\xi },\;\;W^{\left(s\right)}=\mathrm{diag}(w_{1},\ldots ,w_{FM}),$$

where *μ_m_* is the arithmetic mean magnitude of the *m*-th row, and ξ>0 prevents excessive amplification of weak rows. The whitened system is

(15) $${\bar{s}}^{\left(t|s\right)}=W^{\left(s\right)}\Delta s^{\left(t|s\right)},\;\;{\bar{A}}^{\left(s\right)}=W^{\left(s\right)}A^{\left(s\right)},$$

Compute the compact SVD ${\bar{A}}^{\left(s\right)}=U\Sigma V^{H}$ once per gate cycle, with singular values $\sigma_{1}\geq \ldots \geq \sigma_{r}>0$. With κ∈(0,1) denoting the spectral energy keep factor, the truncation order *R* is chosen as the smallest integer satisfying

(16) $$\sum_{l=1}^{R}\sigma_{l}^{2}\geq \kappa \sum_{l=1}^{r}\sigma_{l}^{2}.$$

The TSVD estimate of the gate-referenced increment is

(17) $$\Delta \chi^{\left(t|s\right)}=V_{R}\Sigma_{R}^{-1}U_{R}^{H}{\bar{s}}^{\left(t|s\right)},$$

where $U_{R}$, $\Sigma_{R}$, and $V_{R}$ denote the truncated SVD factors associated with the first *R* singular values. A framewise contrast estimate is finally obtained as

(18) $$\chi^{\left(t\right)}=\chi^{\left(s\right)}+\Delta \chi^{\left(t|s\right)}.$$

### 2.3. Dwell-Based Residual Gating

To assess whether the current gate remains valid, the normalized residual ratio is defined as

(19) $$\eta_{t}=\frac{{\left\|{\bar{s}}^{\left(t|s\right)}-{\bar{A}}^{\left(s\right)}\Delta \chi^{\left(t|s\right)}\right\|}_{2}^{2}}{{\left\|{\bar{s}}^{\left(t|s\right)}\right\|}_{2}^{2}},$$

This quantity measures the normalized mismatch between the whitened state-referenced data and the corresponding gate-linearized response reconstructed from the estimated increment, where $\|\cdot {\|}_{2}$ denotes the Euclidean norm. This residual serves as the gating statistic used to trigger gate refresh.

To avoid frequent refreshes caused by isolated residual excursions, an upper threshold $\eta_{U}$ and a dwell threshold $C_{\mathrm{th}}\in \mathbb{N}$ are introduced. With *c* denoting a counter initialized at zero, the update rule at each frame is

(20) $$c\leftarrow \left\{\begin{array}{ll} c+1, & \eta_{t}\geq \eta_{U}\\ 0, & \eta_{t}<\eta_{U} \end{array}\right.,$$

When $c\geq C_{\mathrm{th}}$, the gate is refreshed by

(21) $$s\leftarrow t,\;\;\chi^{\left(s\right)}\leftarrow \chi^{\left(t\right)},\;\;c\leftarrow 0,$$

followed by recomputing $S_{\mathrm{est}}^{\left(s\right)}(f_{k})$, $A^{\left(s\right)}$, $W^{\left(s\right)}$, and the corresponding SVD factors.

### 2.4. Comparison Methods

Three comparison methods are used later in this paper.

(1) State-based TSVD (S-TSVD) follows the same state-referenced differencing and gate-refresh rule as SR-TSVD but removes row whitening by setting $W^{\left(s\right)}=I$.
(2) Frame-differential whitened TSVD (FD-WTSVD) does not maintain a gate state. It uses adjacent-frame differencing $\Delta S^{\left(t|t-1\right)}(f_{k})=S^{\left(t\right)}(f_{k})-S^{\left(t-1\right)}(f_{k})$, together with the same row-whitened TSVD inversion, but without gate refresh.
(3) The distorted Born iterative method (DBIM) is used as a nonlinear iterative reference. At each iteration, it updates the current background estimate, recomputes the forward fields and linearized operator, and solves for a contrast correction until a stopping criterion or iteration limit is reached.

### 2.5. Algorithm and Parameter

Algorithm 1 summarizes the full SR-TSVD procedure. SR-TSVD reconstructs gate-referenced increments $\Delta \chi^{\left(t|s\right)}$ from state-referenced differential data $\Delta s^{\left(t|s\right)}$ by linearizing the forward model, applying row-wise whitening, and performing energy-truncated TSVD inversion.
**Algorithm 1** State-referenced TSVD with dwell-based residual gating$\mathrm{Require}:\;\mathrm{Baseline}\;\mathrm{background}\;\varepsilon_{\mathrm{bg},0}$$,\;\mathrm{measured}\;\mathrm{multistatic}\;\mathrm{data}\;\{S^{\left(t\right)}(f_{k})\},\;\mathrm{parameters}\;\xi ,\;\kappa ,\;\eta_{U},\;C_{\mathrm{th}}$  1:Initialize s←0, c←0$,\;\mathrm{and}\;\chi^{\left(s\right)}\leftarrow 0$  2:$\mathrm{Compute}\;S_{\mathrm{est}}^{\left(s\right)}(f_{k})$$,\;A^{\left(s\right)}$$,\;W^{\left(s\right)}$$,\;\mathrm{and}\;\mathrm{the}\;\mathrm{truncated}\;\mathrm{SVD}\;\mathrm{of}\;{\bar{A}}^{\left(s\right)}$  3:for each frame t=1,2,… **do**  4:$\mathrm{Form}\;\Delta S^{\left(t|s\right)}(f_{k})=S^{\left(t\right)}(f_{k})-S_{\mathrm{est}}^{\left(s\right)}(f_{k})$  5:$\mathrm{Stack}\;\Delta s^{\left(t|s\right)}$ over all selected frequencies  6:$\mathrm{Compute}\;{\bar{s}}^{\left(t|s\right)}=W^{\left(s\right)}\Delta s^{\left(t|s\right)}$  7:$\mathrm{Solve}\;\Delta \chi^{\left(t|s\right)}$ from (17)  8:$\mathrm{Update}\;\chi^{\left(t\right)}=\chi^{\left(s\right)}+\Delta \chi^{\left(t|s\right)}$  9:$\mathrm{Compute}\;\eta_{t}$ from (19)10:Update *c* according to (20)11:$\mathrm{if}\;c\geq C_{\mathrm{th}}$ **then**12:Refresh the gate state using (21)13:$\mathrm{Recompute}\;S_{\mathrm{est}}^{\left(s\right)}(f_{k})$$,\;A^{\left(s\right)}$$,\;W^{\left(s\right)}$, and the SVD factors14:**end if**15:**end for**

The key parameters are summarized as follows:

Whitening weights $\left\{w_{m}\right\}$ and stabilizer ξ in (14): $w_{m}$ balances the row magnitudes of $A^{\left(s\right)}$ using $\mu_{m}$, while ξ prevents excessive gains when $\mu_{m}$ is small. A smaller ξ strengthens row balancing but may over-amplify weak rows and increase sensitivity to noise, whereas a larger ξ suppresses such amplification at the expense of weaker balancing and reduced structural detail.

Energy keep factor κ in (16): A larger κ retains more singular components and preserves more detail, but it also increases sensitivity to noise and model mismatch, while a smaller κ improves stability at the cost of stronger smoothing.

Gating parameters $\left(\eta_{U},C_{\mathrm{th}}\right)$ in (20) and (21): Smaller values lead to more frequent refreshes and better preserve the validity of the gate-linearized model, but they also increase the number of forward-solver calls. Larger values reduce refresh frequency but may allow mismatch to accumulate within a gate cycle.

## 3. Numerical Results

This section validates SR-TSVD under controlled numerical conditions. The simulations are used to examine its ability to track time-varying hemorrhagic changes, to compare it with S-TSVD, FD-WTSVD, and DBIM, and to assess the effects of array size, frequency diversity, and measurement noise. In all cases, the anatomical background is fixed, and only the hemorrhagic component varies with time.

### 3.1. Simulation Setup and Evaluation Metrics

A two-dimensional frequency-domain finite-element method (FEM) model under a transverse magnetic (TM) scalar formulation was adopted. This two-dimensional setting was chosen as a computationally efficient framework for mechanism validation because a full three-dimensional implementation of SR-TSVD would require a substantially higher cost for repeated gate-state updates and linearized operator rebuilding.

The computational domain was a circular region with a radius of 15 cm, containing a head-like cross-section immersed in a coupling medium and terminated by a perfectly matched layer (PML). A uniform circular array with *N* = 16 measurement positions and a radius of 100 mm was used. A 20 mm thick PML was applied near the outer boundary to suppress spurious reflections.

**Figure 1:**
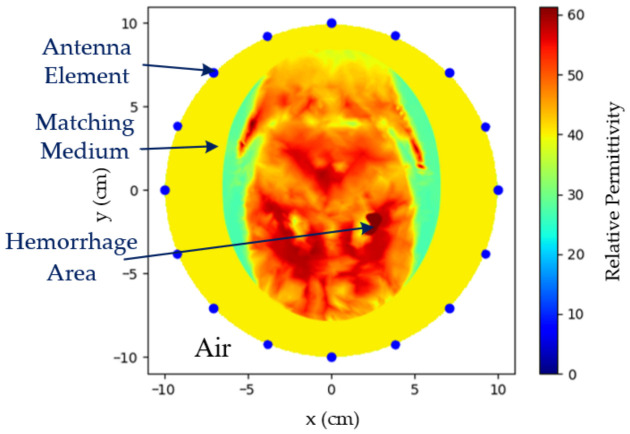
Two-dimensional FEM simulation configuration and phantom layout. A 16-element circular array (radius 10 cm) surrounds the head-like phantom. The coupling medium fills the gap between the array and the head. The cross-section illustrates the relative permittivity distribution of the coupling medium, skull, brain tissue, and hemorrhagic inclusions, whereas only the hemorrhagic inclusion changes across frames.

The brain region was represented by an ellipse with a major axis of 16 cm and a minor axis of 13 cm. Within this model, the hemorrhagic region and the coupling medium were assigned relative permitivities of 60 and 40, and conductivities of 1.5 S/m and 0.5 S/m, respectively, whereas all other tissues were incorporated into the background. The detailed medium distribution is shown in Figure 1. Each array position was treated as a point transmitter, and the complex electric field was sampled at the remaining receiver positions.

For a transmitting source *i*, receiver *j*, frame *t*, and frequency *f*, the off-diagonal response entry was defined as

(22) $$S_{ij}^{\left(t\right)}(f)=\frac{E_{j}^{\left(t\right)}(r_{j}|i,f)-E_{j}^{\mathrm{bg}}(r_{j}|i,f)}{E_{j}^{\mathrm{bg}}(r_{j}|i,f)},\;\;i\neq j,$$

where $E_{j}^{\left(t\right)}(r_{j}|i,f)$ denotes the complex field sampled at the *j*-th receiver position under excitation from source *i*, and $E_{j}^{\mathrm{bg}}(r_{j}|i,f)$ denotes the corresponding background-field sample from the fixed anatomical background. All diagonal terms were discarded, and the resulting off-diagonal matrix served as the two-dimensional surrogate of the multistatic scattering matrix used in reconstruction.

The finite-element mesh was generated so that the maximum element size was approximately one-tenth of the minimum wavelength in the computational domain. The region of interest (ROI) used for inversion was discretized into 100 × 100 pixels, corresponding to a physical size of 200 mm × 200 mm.

The main simulations used three frequencies, f∈{0.95, 1.00, 1.05} GHz, which is consistent with the narrow-band assumption adopted in Section 2. To examine the effect of frequency diversity, additional comparisons were carried out using only 1.00 GHz and using five frequencies: 0.95/0.975/1.00/1.025/1.05 GHz. Complex additive white Gaussian noise was added to the simulated response matrix to set the signal-to-noise ratio (SNR) to 30 dB.

Four dynamic scenarios were considered: single-lesion growth (a1), multi-lesion growth (a2), single-lesion regression (b1), and multi-lesion regression (b2). Each scenario consisted of nine frames and was repeated in four independent trials. For the growth sequences, the lesion radius increased monotonically across frames, whereas for the regression sequences, the corresponding growth sequence was reversed in time.

Four imaging schemes were implemented:

(1) For SR-TSVD, the parameters were fixed as $\xi =10^{-4}$, κ=0.95, $\eta_{U}=0.30$, and $C_{\mathrm{th}}=2$. These values were chosen based on coarse parameter sweeps to balance reconstruction accuracy, refresh frequency, and computational cost. In particular, the choice of ξ was further examined through an additional whitening-parameter comparison reported in Section 3.3.3.
(2) S-TSVD used the same state-referenced differencing and gate-refresh rule, but without whitening. Although S-TSVD and SR-TSVD use the same gate-refresh rule, the dwell counter *c* may evolve differently because SR-TSVD uses a row-whitened residual.
(3) FD-WTSVD used adjacent-frame differencing together with the same TSVD inversion, but without an internal gate state.
(4) For DBIM, the baseline background was used as the initialization, the maximum iteration number was set to 10, the relative residual stopping threshold was set to $10^{-1}$, the regularization parameter was set to $10^{-2}$, and the relaxation factor was set to 0.8.

For S-TSVD and SR-TSVD, the increment at frame *t* was defined as the difference between the current reconstruction and the gate state. For FD-WTSVD, the increment was defined with respect to the previous frame.

To quantitatively assess reconstruction accuracy, the mean squared error (MSE) and structural similarity index measure (SSIM) are used as image quality metrics. All metrics are computed within the ROI across methods. Let $\hat{\chi }\in {\mathbb{C}}^{P}$ and $\chi_{\mathrm{ref}}\in {\mathbb{C}}^{P}$ denote the reconstructed and reference complex maps, respectively, where *P* is the number of ROI pixels. The MSE is defined as $\mathrm{MSE}=\frac{1}{P}\sum_{p=1}^{P}{\left|{\hat{\chi }}_{p}-\chi_{\mathrm{ref},p}\right|}^{2},$ and the corresponding magnitude-image structural similarity score is $\mathrm{SSIM}\;(\left|\hat{\chi }\right|,\left|\chi_{\mathrm{ref}}\right|)$.

### 3.2. Results of Dynamic Tracking

This subsection focuses on dynamic changes in hemorrhage size and number. For a fair comparison, the non-state baseline (FD-WTSVD) operates on frame-differential measurements formed by differencing scattering parameters with respect to the previous frame. In contrast, the state-based methods (S-TSVD and SR-TSVD) consume raw measurements at each frame and perform internal state-referenced differencing via the gated background update.

**Figure 2:**
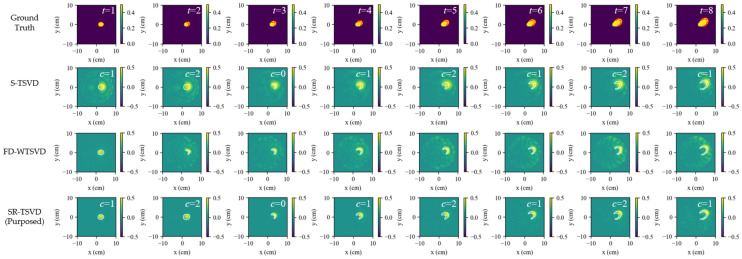
Dynamic tracking results for scenario a1 (single-lesion growth). Rows show ground truth and reconstructions by S-TSVD, FD-WTSVD, and SR-TSVD. Columns correspond to successive frames. The gate counter *c* is annotated in each frame. The first row shows the ground truth as the real part of the total contrast map relative to the baseline background, where the red contour indicates the region that changed relative to the previous frame. The reconstruction results are displayed in the real part of the contrast increment, with the white contour the true change boundary relative to the reference frame.

Figure 2 compares the ground truth, S-TSVD, FD-WTSVD, and SR-TSVD in scenario (a1), where columns indicate different frames *t*. The first row shows the ground truth as the real part of the total contrast map relative to the baseline background, whereas the reconstruction results of S-TSVD, FD-WTSVD, and SR-TSVD are displayed as the real part of contrast increments relative to the corresponding reference state used by each method. In the ground-truth maps, the red contour marks the newly increased region relative to the reference frame, and the blue contour marks the reduced region. In each reconstruction, the white contour indicates the true difference boundary between the current frame and the reference frame.

The gate counter *c* is shown at the upper-right corner of each reconstruction, which triggers a background update when it reaches the dwell threshold. For the present case, S-TSVD and SR-TSVD triggered gate refresh at the same frames. However, their refresh instants need not be identical, because the residual statistic is evaluated from non-whitened and whitened systems, respectively. S-TSVD maintains a gate state, allowing small changes to remain referenced to the same background over multiple frames. This improves temporal continuity and helps preserve weak accumulated variations. Without row whitening, however, channel imbalance is not explicitly compensated, so the reconstructed maps tend to show weaker structural definition, with less distinct fine detail and less accurate boundary delineation. FD-WTSVD instead reconstructs direct adjacent-frame differences using a fixed kernel. Its row whitening increases sensitivity to local frame-to-frame changes and makes small structures more visible, although this also increases susceptibility to fluctuation-induced artifacts. SR-TSVD combines the advantages of both strategies. It retains the temporal stability associated with state referencing while improving structural detail through whitening, yielding the most balanced reconstruction among the three linear methods in this case.

**Figure 3:**
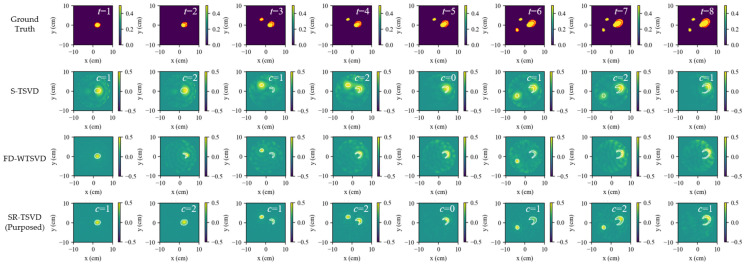
Dynamic tracking results for scenario a2 (multi-lesion growth). Rows show ground truth and reconstructions by S-TSVD, FD-WTSVD, and SR-TSVD. Columns correspond to successive frames. The red contour in ground truth indicates the region that changed relative to the previous frame, while the white contour in each reconstructed subfigure indicates the true difference boundary between the current frame and the reference frame.

Figure 3 shows scenario (a2), where both the number and size of inclusions increase with *t*. Multiple evolving targets slightly degrade accuracy for all methods compared with (a1). S-TSVD still benefits from the maintained gate state and, therefore, remains more temporally coherent than FD-WTSVD, especially when two inclusions evolve concurrently. FD-WTSVD is more responsive to local frame-to-frame variations and can emphasize weak changes more clearly, but the same sensitivity also leads to stronger spurious responses when multiple targets evolve simultaneously. SR-TSVD remains comparatively robust, showing only minor distortions while preserving lesion localization.

**Figure 4:**
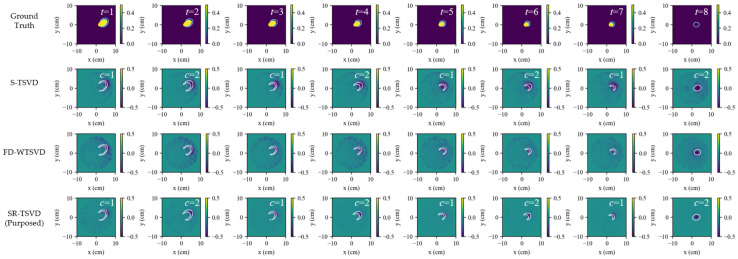
Dynamic tracking results for scenario b1 (single-lesion regression). Rows show ground truth and reconstructions by S-TSVD, FD-WTSVD, and SR-TSVD. Columns correspond to successive frames. The blue contour in ground truth indicates the region that changed relative to the previous frame, while the white contour in each reconstructed subfigure indicates the true difference boundary between the current frame and the reference frame.

**Figure 5:**
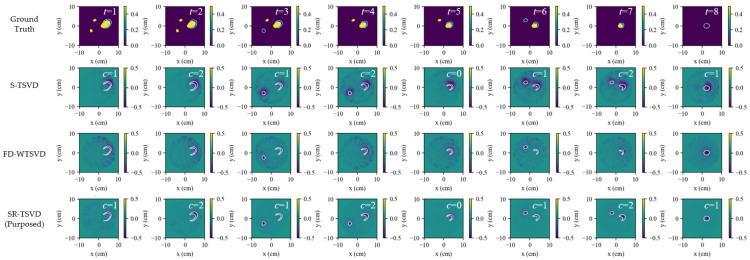
Dynamic tracking results for scenario b2 (multi-lesion regression). Rows show ground truth and reconstructions by S-TSVD, FD-WTSVD, and SR-TSVD. Columns correspond to successive frames. The blue contour in ground truth indicates the region that changed relative to the previous frame, while the white contour in each reconstructed subfigure indicates the true difference boundary between the current frame and the reference frame.

The group-b sequences are generated by time reversing the scattering sequences from group-a. Figure 4 and Figure 5 show the scenarios for absorption and disappearance of inclusions. The same relative behavior is observed in these sequences. S-TSVD benefits from the gate-state reference and, therefore, remains less sensitive to isolated frame-to-frame fluctuations, although its structural detail is still comparatively weak. FD-WTSVD responds more strongly to local inter-frame changes, which improves visual sharpness in some regions but also increases the likelihood of fluctuation-induced artifacts. SR-TSVD still yields the most balanced result by combining the accumulated sensitivity of state referencing with the detail enhancement provided by whitening.

**Figure 6:**
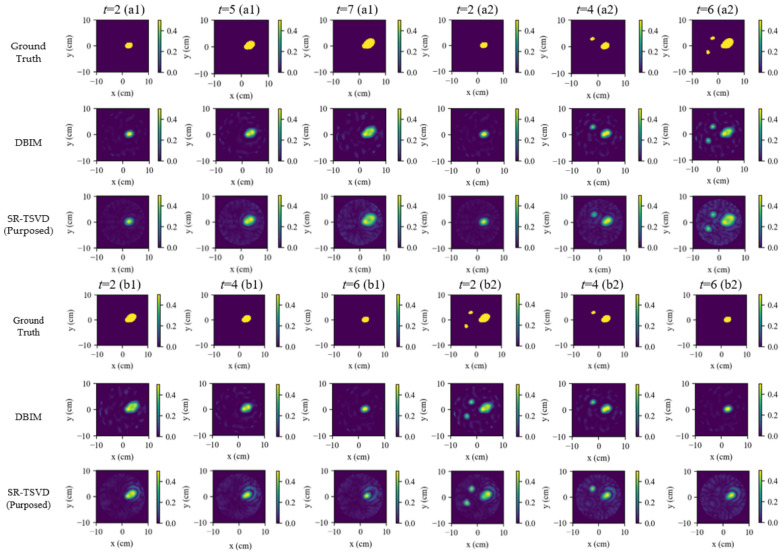
Gate refreshes in numerical experiments. Results are shown only at frames where SR-TSVD triggers a gate refresh. Each column corresponds to one update frame, and the panels compare the refreshed SR-TSVD gate state and the DBIM reconstruction under the same scenario.

The gate-refresh behavior of SR-TSVD is further examined in Figure 6, which shows the frames with a gate refresh. At these frames, the refreshed gate state is compared with a DBIM reconstruction under the same measurement setting. The maps in this figure are displayed in contrast. This comparison assesses whether the refreshed gate state remains consistent with a quasi-static iterative reference. Across the tested cases, the refreshed gate states follow the main lesion structures produced by DBIM while avoiding nonlinear re-solving at every frame.

### 3.3. Quantitative Comparison

#### 3.3.1. Overall Performance and Cost

Table 1 summarizes the quantitative performance over the four dynamic scenarios. For each method and scenario, the MSE and SSIM were first computed on the framewise increment maps, then averaged over all frames within each trial, and finally summarized across the four independent trials.

Overall, the three TSVD-type methods exhibit a clear performance ordering. S-TSVD benefits from state referencing and, therefore, maintains better temporal continuity than FD-WTSVD, but the absence of whitening limits its ability to recover fine structural detail. FD-WTSVD is more responsive to local frame-to-frame changes because of row whitening, yet this increased sensitivity also leads to more visible artifacts. SR-TSVD combines the two mechanisms and, therefore, provides the most balanced dynamic-tracking performance among the three linear methods.

Table 2 reports the full-lesion comparison on refresh frames, where the refreshed gate state of SR-TSVD is compared with DBIM under matched settings. DBIM serves here as a nonlinear iterative reference. By repeatedly updating the background and re-solving the forward problem, it provides a better approximation to the nonlinear scattering generated by complex target structures. As a result, DBIM generally produces cleaner full-lesion reconstructions with stronger suppression of background artifacts and noise. In contrast, SR-TSVD is designed to maintain an evolving gate state with much lower computational cost.

In addition to image quality metrics, Table 3 reports the computational cost in the numerical study. For S-TSVD and SR-TSVD, the runtime is separated into non-refresh frames and refresh frames. Non-refresh frames involve only the TSVD projection using the current operator, whereas refresh frames additionally require rebuilding the gate-dependent forward fields, linearized operator, whitening weights, and truncated SVD factors. FD-WTSVD has no refresh frames because it uses a fixed baseline operator. The non-refresh cost remains at the millisecond level for all TSVD-type solvers, whereas refresh frames incur second-level costs. Compared with the refreshed gate-state update of SR-TSVD, single-frame DBIM reconstruction requires substantially more computation, typically more than eight times longer in the present setting.

This difference highlights the distinct use cases of the two methods. SR-TSVD is better suited to real-time tracking, while DBIM is more appropriate when higher reconstruction fidelity is prioritized over speed.

**Figure 7:**
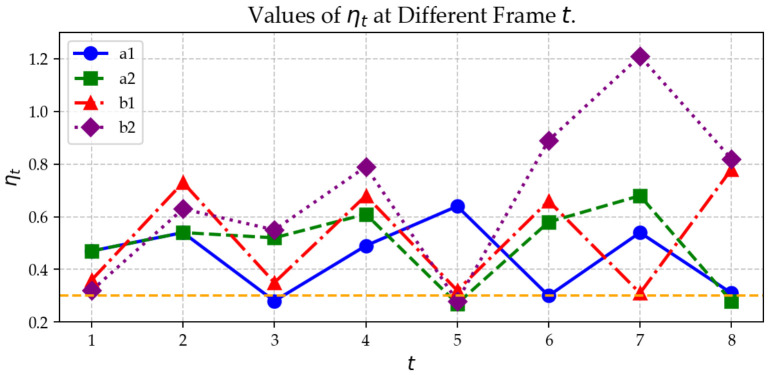
Per-frame gating statistic $\eta_{t}$ in numerical experiments. The dashed line marks the threshold $\eta_{U}$. When $\eta_{t}\geq \eta_{U}$, the counter *c* is incremented; a gate refresh is triggered when $c\geq C_{\mathrm{th}}$, after which $\eta_{t}$ typically drops.

To make the gating behavior explicit, per-frame values of the gating statistic $\eta_{t}$ for all four experiments are plotted in Figure 7, where the dashed line marks threshold $\eta_{U}$. The hemorrhagic region evolves slowly over time, making $\eta_{t}$ remain nonzero, and the SR-TSVD gate typically triggers a background update every two to three frames. At update frames, $\eta_{t}$ drops markedly, indicating successful assimilation of the updated gate state.

#### 3.3.2. Effects of Array Size and Frequency Diversity

To bridge the gap between the 16-element numerical study and the later eight-element hardware prototype, the effects of array size and frequency diversity were examined under otherwise identical conditions. These two factors are closely related in sparse-array dynamic imaging: the number of antennas determines the available angular sampling, whereas the number of frequencies affects the degree of spectral diversity available to compensate for limited spatial sampling.

**Figure 8:**
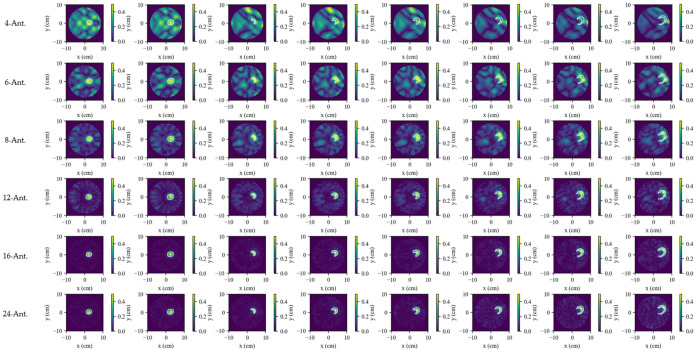
Influence of antenna count on SR-TSVD dynamic monitoring performance. The white contour in each reconstructed subfigure indicates the true difference boundary between the current frame and the reference frame.

The effect of antenna count is illustrated in Figure 8 and quantified in Table 4, where the SR-TSVD results are compared for four, six, eight, 12, 16, and 24 antennas using the single-lesion growth sequence. The results show that spatial sampling is the dominant factor in dynamic monitoring. With only four or six antennas, the reconstructions are severely degraded and do not provide reliable lesion tracking under the present setting. At eight antennas, the main lesion evolution is recovered, although a noticeable gap remains relative to denser arrays. The transition from four to eight antennas is seen to be more important than the transition from 16 to 24 antennas, indicating diminishing returns once the spatial sampling becomes sufficiently dense.

**Figure 9:**
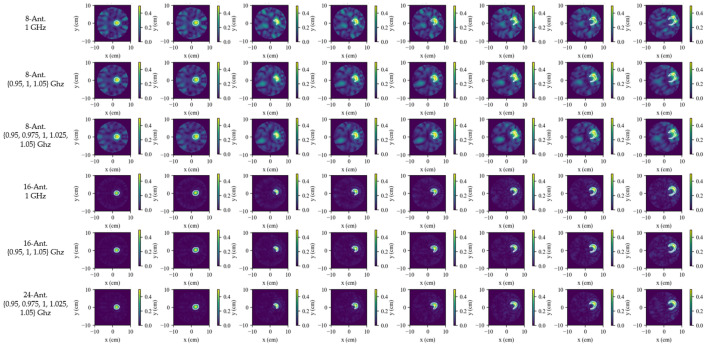
Influence of frequency diversity under different antenna counts. The white contour in each reconstructed subfigure indicates the true difference boundary between the current frame and the reference frame.

The influence of frequency diversity is then examined in Figure 9 and Table 5. Three settings are compared: single-frequency inversion at 1.00 GHz, three-frequency inversion at 0.95/1.00/1.05 GHz, and five-frequency inversion at 0.95/0.975/1.00/1.025/1.05 GHz. For sparse arrays, frequency diversity provides a clear benefit. In particular, at eight antennas, the multi-frequency settings outperform the single-frequency case by a clear margin. However, the difference between the three-frequency and five-frequency settings remains small, indicating diminishing returns within the present narrow band. For the 16-antenna array, the difference between single-frequency and multi-frequency inversion is much less pronounced.

The runtime analysis shows a clearer trade-off. As the number of frequencies increases, the refresh-frame reconstruction time rises substantially for both antenna configurations. This increase is much more pronounced than the corresponding gain in image quality when moving from three frequencies to five. These results show that both angular sampling and spectral diversity affect dynamic-monitoring performance, although angular sampling remains the dominant factor. For the sparse eight-antenna setting considered in the later hardware study, the three-frequency configuration provides the most suitable compromise between reconstruction quality and computational cost.

#### 3.3.3. Effect of the Whitening Stabilizer

To examine the effect of the whitening stabilizer in (14), an additional comparison was carried out using FD-WTSVD on the single-lesion growth sequence (scenario a1). FD-WTSVD was chosen here because it uses the whitening operation but does not involve a maintained gate state, which makes the influence of *ξ* easier to observe directly. All other simulation settings were kept the same as in Section 3.1, and only the stabilizer value was changed.

The tested range of *ξ* was selected according to the magnitude distribution of the linearized operator. Under the present simulation setting, the absolute values of the entries of $A^{\left(s\right)}$ span approximately from $6.97\times 10^{-6}$ to $7.87\times 10^{-3}$, with a median value of about $1.34\times 10^{-4}$. This indicates that a practical stabilizer should lie near the $10^{-4}$ scale so that it can suppress excessive amplification of weak rows without eliminating the balancing effect of whitening. To illustrate the influence of under- and over-regularization more clearly, three logarithmically spaced values were tested, namely, $\xi =10^{-3}$, $\xi =10^{-4}$, and $\xi =10^{-5}$.

**Figure 10:**
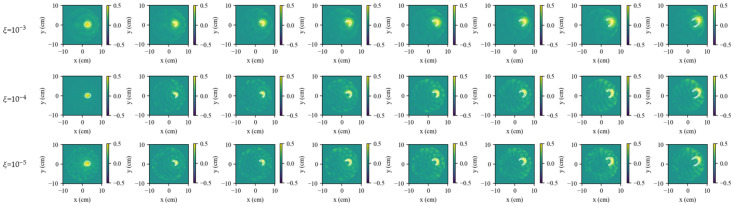
Effect of the whitening stabilizer *ξ* on FD-WTSVD for scenario a1. The white contour indicates the true difference boundary.

The results are shown in Figure 10. When $\xi =10^{-3}$, the whitening effect is relatively weak, so dominant rows are not sufficiently balanced, and the reconstructed maps tend to lose structural detail. When $\xi =10^{-5}$, weak rows are amplified more aggressively, which leads to more visible noise and fluctuation-induced artifacts. Among the tested values, $\xi =10^{-4}$ provides the best overall balance between structural detail and robustness and is, therefore, used in the subsequent experiments.

These observations are consistent with the role of *ξ*. A larger stabilizer suppresses excessive gains but weakens the balancing effect of whitening, whereas a smaller stabilizer strengthens balancing at the cost of greater sensitivity to noise and weak-row amplification. Under the present setting, $\xi =10^{-4}$ provides the most suitable compromise between structural definition and robustness.

#### 3.3.4. Noise Robustness

In the previous simulations, the SNR was set to 30 dB. To examine robustness against measurement noise, a representative frame *t* = 6 from scenario a1 was re-imaged at four noise levels: 20 dB, 15 dB, 10 dB, and 5 dB.

**Figure 11:**
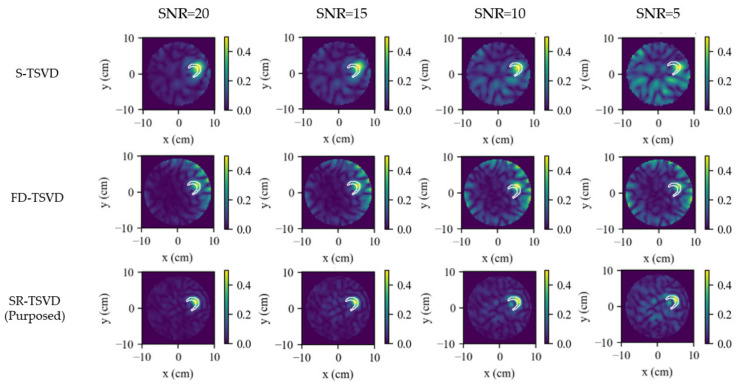
Noise robustness evaluation on scenario a1 at frame *t* = 6 under different SNR levels (20, 15, 10, and 5 dB). Rows show reconstructions by FD-WTSVD, S-TSVD, and SR-TSVD under the same measurement settings. The white contour in each reconstructed subfigure indicates the true difference boundary.

As shown in Figure 11, image quality deteriorates as the SNR decreases. All methods exhibit a growing artifact burden and noticeable localization bias at low SNRs. SR-TSVD benefits from gated state referencing and whitening, and its reconstructions remain superior across the tested SNR range. FD-WTSVD shows a clearer advantage over S-TSVD as the SNR decreases because whitening reduces channel dominance and stabilizes TSVD inversion. S-TSVD, while benefiting from state referencing, degrades more noticeably without whitening when noise dominates the differential data. Overall, FD-WTSVD improves stability through whitening but lacks state refresh, which can lead to increased mismatch over long sequences. In contrast, SR-TSVD achieves superior performance in both detail preservation and noise robustness by combining state-referenced gating with row-whitened TSVD.

## 4. Hardware Phantom Experiments

To further evaluate SR-TSVD under practical measurement conditions, dynamic phantom experiments were carried out using a portable microwave acquisition platform. Compared with the numerical study, the hardware experiments introduce additional mismatch from antenna fabrication tolerances, switching-dependent channel variability, and assembly-induced inconsistency. This section examines whether the monitoring-oriented reconstruction strategy remains effective under an eight-element prototype and realistic measurement constraints.

### 4.1. Experimental Setup

**Figure 12:**
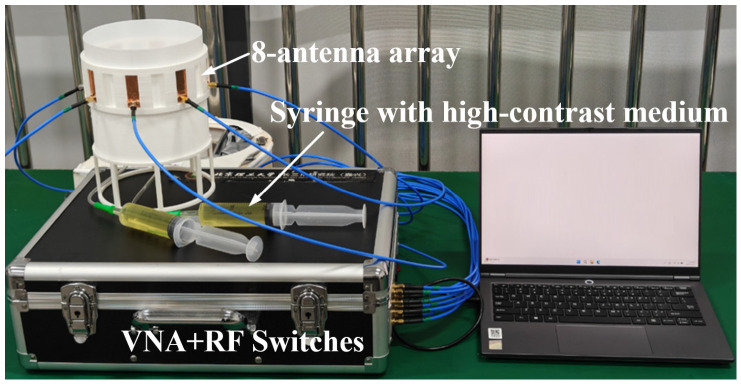
Hardware system architecture for phantom experiments, including host computer, VNA, RF switch matrix, 8-element circular antenna array, and target container.

The hardware platform comprises a host computer, a vector network analyzer (VNA, N9915A, Keysight Technologies), a radio-frequency (RF) switch matrix, an eight-element circular antenna array, and a cylindrical target container, as illustrated in Figure 12. The VNA performs a 0.5–1.5 GHz sweep with 101 frequency points and an intermediate-frequency (IF) bandwidth of 100 Hz to obtain S-parameters. The RF switch matrix routes a two-port VNA across the array so that all inter-element S-parameters can be measured without cable re-patching. Both the VNA and the RF switch matrix are integrated within a portable aluminum briefcase. Eight microstrip patch antennas [36] are uniformly distributed on a circle of 10 cm radius and are kept in firm contact with the container wall to maintain impedance matching and radiation efficiency.

An eight-antenna array was used instead of the 16-antenna array adopted in simulations. The sparser array simplifies the switching network, reduces insertion loss and crosstalk in the RF chain, mitigates strong mutual coupling among closely packed elements, and shortens the acquisition cycle for dynamic monitoring. These practical constraints reflect a compact monitoring-oriented prototype, at the cost of reduced angular sampling compared with the 16-antenna simulation setting.

**Figure 13:**
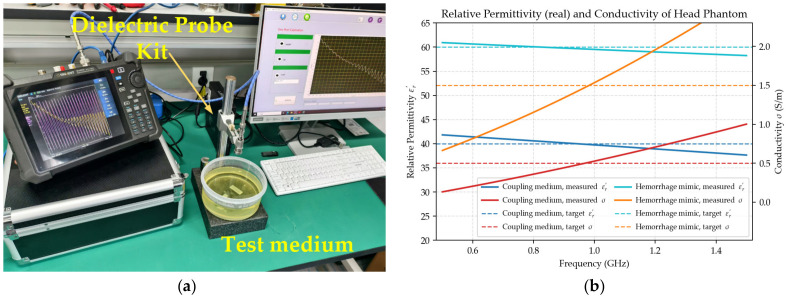
Dielectric characterization of the phantom mixtures. (**a**) One-port coaxial-probe measurement setup; (**b**) measured complex relative permittivity (real part and conductivity-equivalent loss) over the operating band.

To approximate in vivo dielectric conditions, a simplified head phantom based on an aqueous polyvinylpyrrolidone (PVP)–NaCl mixture was prepared. The phantom consists of a coupling medium that mimics brain tissue and high-contrast inclusions that emulate hemorrhage. The coupling medium targets $\varepsilon_{r}$ ≈ 40 and *σ* ≈ 0.5 S/m at 1 GHz, achieved with 838.8 g of PVP per kg of H_2_O. The hemorrhage mimic targets $\varepsilon_{r}$ ≈ 60 and *σ* ≈ 1.5 S/m at 1 GHz, obtained by mixing 310.7 g of PVP + 13.2 g of NaCl per kg of H_2_O. The complex permittivity of the mixtures was characterized using a one-port coaxial probe over the operating band with a spectrum analyzer (SHA851A, SIGLENT Technologies), as shown in Figure 13 [37,38].

**Figure 14:**
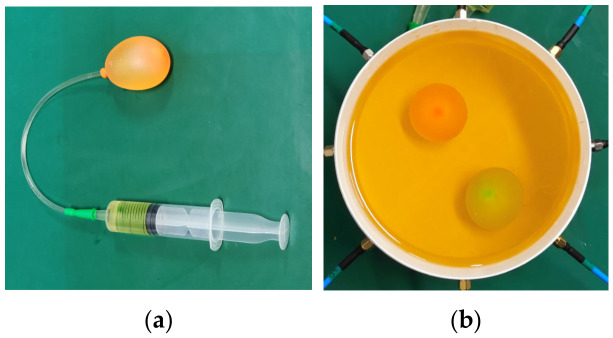
Experimental phantom and inclusion control. (**a**) Syringe connected to a balloon via flexible tubing; (**b**) target container with bottom openings sealed to prevent leakage.

To induce controlled changes in lesion size and number, balloons filled with the hemorrhage mimic served as inclusions (Figure 14). Each balloon was connected to a syringe via soft tubing that entered through a sealed opening at the container’s bottom. The balloon volume was adjusted to predefined targets by displacing the plunger. During each frame, the active balloon(s) were adjusted to lie approximately in the imaging plane defined by the phase centers of the eight patch elements. When a second balloon was introduced, it was initially fixed near the bottom of the container and became active only after filling, after which buoyancy brought it into the imaging plane. For each frame, the intended inclusion center positions and injected volumes were recorded for constructing frame-wise ground-truth masks.

### 4.2. System Calibration and Data Acquisition

Each switched transmit–receive path was pre-calibrated to improve the repeatability of the measured S-parameters. An electronic calibration (E-Cal) module was connected to the two VNA ports feeding the transmit and receive channels. For each switched route, the host computer selected the corresponding RF path, performed a two-port E-Cal, and saved the resulting calibration state. During acquisition, after switching to a given route, the host loaded the corresponding saved calibration state, inserted a 0.5 s settling delay, and then triggered a sweep. For each frame, two sweeps were recorded and averaged to improve the SNR. This procedure is, therefore, a path-wise two-port calibration rather than a simultaneous full N-port calibration. Beyond the path-wise two-port calibration, a standard cylindrical target placed at the array center was used to determine a frequency-wise complex scaling factor between measurement and simulation.

Although the VNA sweep covers 0.5–1.5 GHz, imaging was performed using three frequency points f∈{0.95,1.00,1.05} GHz to match the simulation study and to form a compact multi-frequency data vector for inversion. This choice follows the numerical study and remains consistent with the narrow-band approximation adopted in Section 3.3.2. For the eight-antenna setting, introducing a small number of nearby frequencies improves reconstruction relative to the single-frequency case, while adding further frequency points provides only marginal gains at the cost of increased computation. At the same time, the narrow-band setting helps limit the influence of dispersion on the measured data and the reconstruction model. The full-band sweep was, therefore, retained for measurement consistency and dielectric characterization, whereas all reconstructions used the same three-frequency subset.

A complete multistatic measurement required approximately 84.5 s, including switching, calibration-state recall, settling delay, and repeated sweeps. Since the hardware experiment does not strictly satisfy the two-dimensional assumption used in simulation, some discrepancy in the tracking results is unavoidable. Therefore, the main purpose of this section is to verify the consistency of the proposed method between the simulated and real measurement systems, rather than to seek exact agreement in reconstruction details.

A dynamic sequence with two potential hemorrhagic sites was acquired and organized into three stages:

(1) *t* = 0 is hemorrhage-free, frames *t* = 1 and *t* = 2 cover the onset and growth of a single inclusion.
(2) At *t* = 3, a second inclusion is introduced, while the first begins regression.
(3) At *t* = 6, the first inclusion has fully regressed, and the second begins regression.

Table 6 lists the recorded inclusion centers and injected volumes for all frames. To better connect the 3-D balloon control to the 2-D imaging model, the table includes the equivalent spherical diameter used to construct the ground truth.

### 4.3. Results of Hardware Experiments

All methods were evaluated on the same measured data with matched reconstruction ROI and parameters. The compared algorithms are consistent with the numerical study and include S-TSVD, FD-WTSVD, DBIM, and SR-TSVD. For FD-WTSVD, the input at each frame was the adjacent-frame difference $S^{\left(t\right)}-S^{\left(t-1\right)}$ to emphasize dynamic changes without maintaining an internal state. In contrast, S-TSVD and SR-TSVD operated on raw measurements at each frame and performed internal state-referenced differencing via the gated background update. DBIM was used only as an iterative reference at frames where SR-TSVD triggered a gate refresh, enabling a matched comparison of background-state consistency.

Relative to the numerical study, the hardware experiments are more sensitive to antenna fabrication tolerances and switching-induced amplitude/phase inconsistency, which reduces the effective differential SNR. In addition, the reduced physical sampling lowers angular diversity and degrades the attainable reconstruction fidelity. To improve sensitivity to rapidly varying sequences, the hardware study used a lower gating threshold, $\eta_{U}=0.12$, together with $C_{\mathrm{th}}=2$, so that the gate state could be refreshed before mismatch accumulated excessively.

**Figure 15:**
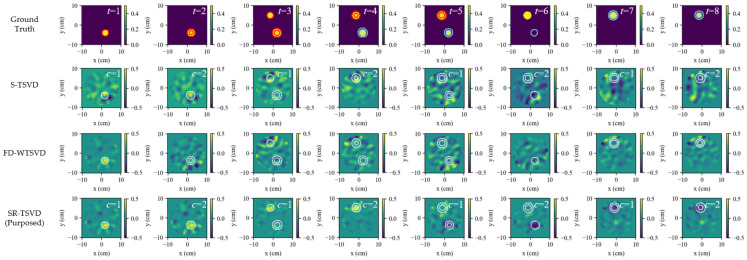
Hardware phantom results for dynamic tracking. Columns correspond to frames. Rows show ground-truth maps constructed from projected areas, and reconstructions by S-TSVD, FD-WTSVD, and SR-TSVD. In the ground-truth maps, the red contour indicates the newly increased region and the blue contour indicates the reduced region relative to the previous frame. The white contour in each reconstructed subfigure indicates the true difference boundary between the current frame and the reference frame.

The dynamic tracking results are shown in Figure 15. The first row presents the 2-D ground truth constructed from the recorded volumes and centers. The following rows show the reconstructions obtained by S-TSVD, FD-WTSVD, and SR-TSVD, respectively. In the ground-truth maps, the red contour marks the newly increased region, and the blue contour marks the reduced region relative to the reference frame. In each reconstructed subfigure, the white contour indicates the true difference boundary between the current frame and the reference frame.

The hardware results follow the same general tendency as the numerical study, while the reduced angular sampling of the eight-antenna system and the additional measurement uncertainties make the differences among methods more pronounced. S-TSVD remains relatively stable over time, but its structural detail is weaker. FD-WTSVD is more responsive to local frame-to-frame changes, yet both baseline methods still exhibit a clear gap relative to SR-TSVD. SR-TSVD again provides the most balanced reconstruction, preserving the main dynamic evolution with better overall quality than the two baseline methods.

When two inclusions evolve concurrently, SR-TSVD preserves lesion localization more reliably than the two TSVD baselines. Nevertheless, within a single gate cycle, a relatively small change can still be masked by a larger simultaneous variation, which is consistent with the state-referenced formulation. The dominant residual component is preferentially captured, whereas a weaker concurrent change may become less visible before the next gate refresh. A similar effect appears when the target evolution is non-monotonic within one gate cycle. If an inclusion first grows and then shrinks before the gate is refreshed, part of that intermediate decrement may be absorbed into the same gate state rather than reconstructed as a distinct negative change. Once a refresh is triggered, the accumulated mismatch is incorporated into the updated gate state, and the reconstruction consistency is recovered.

As shown in Table 7, SR-TSVD achieves the lowest MSE and the highest SSIM among the three linear methods. S-TSVD retains the stabilizing effect of state referencing, but its structural detail is weaker. FD-WTSVD shows a slightly higher SSIM than S-TSVD, yet both baseline methods still exhibit a clear gap relative to SR-TSVD. By combining state referencing with row whitening, SR-TSVD provides the best overall balance under the present hardware conditions.

**Figure 16:**
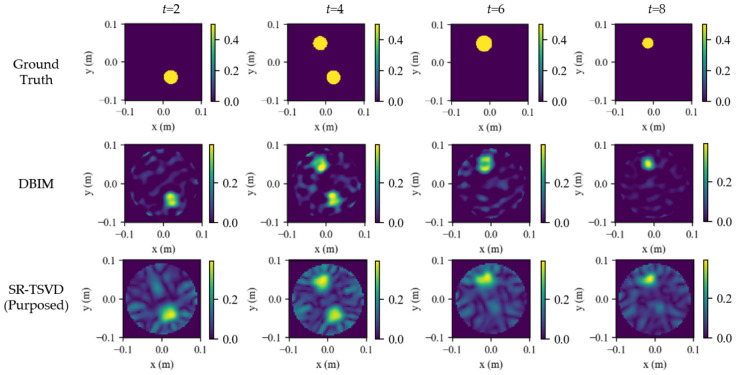
Gate refreshes in hardware experiments. Results are shown only at frames where SR-TSVD triggers a gate refresh. Each column corresponds to one refresh frame, comparing the SR-TSVD updated gate state and the DBIM reconstruction under the same measurement settings.

The gate-refresh behavior is further illustrated in Figure 16. DBIM still provides the sharpest contours as a quasi-static iterative reference, but the refreshed gate state follows the main lesion extent and location closely while avoiding iterative re-solving at every frame. Table 8 further summarizes the full-lesion comparison between the refreshed gate state of SR-TSVD and DBIM over all refresh frames. The hardware results show the same overall trend as the numerical study. DBIM still provides slightly cleaner background regions and stronger artifact suppression, whereas SR-TSVD maintains a lower computational cost and preserves the main dynamic evolution through its gate-state update. Because the hardware system uses only eight antennas, the available angular sampling is sparser than in the 16-antenna numerical study, and the overall reconstruction quality is correspondingly lower. Under this condition, both DBIM and SR-TSVD exhibit some residual artifacts. Nevertheless, the maintained gate state still enables SR-TSVD to track lesion evolution reliably over time, which is consistent with its monitoring-oriented design.

Table 9 reports the reconstruction cost. The non-refresh time remains at the millisecond level for all TSVD-type solvers, while refresh frames incur second-level costs because the gate-dependent fields and system matrices must be rebuilt. Compared with the numerical study, the measured reconstruction times are lower because the hardware prototype uses only eight antennas rather than 16, which reduces the number of multistatic channels and the associated matrix-construction cost. Compared with the refreshed gate-state update of SR-TSVD, single-frame DBIM reconstruction requires substantially more computation. SR-TSVD is, therefore, better suited to real-time tracking, whereas DBIM is more appropriate when higher reconstruction fidelity is prioritized over speed.

Overall, the hardware study confirms the same mechanism suggested by the numerical experiments. State referencing improves temporal consistency by maintaining a stable evolving reference, whereas row whitening enhances sensitivity to local changes but can also amplify fluctuation-induced artifacts when used alone. By combining these two elements, SR-TSVD provides a more balanced reconstruction than either S-TSVD or FD-WTSVD. DBIM remains a valuable quasi-static iterative reference at gate-refresh frames, but SR-TSVD is intended for longitudinal dynamic monitoring, where stable tracking at moderate computational cost is more important than maximizing the sharpness of a single frame.

## 5. Discussion

This study investigated SR-TSVD for dynamic microwave monitoring of intracranial hemorrhage under sparse multistatic sampling. Across the numerical and hardware results, the main conclusion is not merely that SR-TSVD produces cleaner reconstructions than the two TSVD-type baselines, but that it provides a more suitable operating point for dynamic monitoring. By maintaining a gate state and reconstructing only a small state-referenced increment at each frame, SR-TSVD keeps the inversion lightweight on non-refresh frames while still allowing the gate state to be refreshed when mismatch accumulates.

A central reason for this behavior is the combination of state referencing and row whitening. State-referenced differencing reduces the reliance on direct adjacent-frame subtraction and helps prevent a previously evolved lesion region from being repeatedly interpreted as a new change. This becomes especially important when multiple inclusions evolve simultaneously, because direct frame differencing is more likely to mix the evolution of one target with the apparent change of another. The gate-state formulation instead constrains the reconstruction to the current state-referenced increment and, therefore, improves temporal consistency. Row whitening plays a complementary role. In the hardware setting, switching-dependent channel variability and assembly tolerances lead to nonuniform channel strength. Whitening suppresses the dominance of a small subset of strong channels and makes the inversion less sensitive to route-dependent imbalance. The resulting improvement is, therefore, not only numerical but also practical, because it addresses a major source of instability in repeated measurements. The additional stabilizer study further clarifies this trade-off. When the whitening stabilizer is chosen too large relative to the operator magnitude scale, the balancing effect becomes weak, and the recovered structural detail remains limited. When it is chosen too small, weak rows are over-amplified, and the inversion becomes more sensitive to noise and fluctuation-induced artifacts. The present results, therefore, support the use of a stabilizer at the order of $10^{-4}$ as a practical compromise under the tested setting. Taken together, these two mechanisms explain why SR-TSVD remains more stable than S-TSVD and FD-WTSVD in both the numerical and hardware experiments.

The comparison with DBIM highlights the importance of selecting reconstruction algorithms in accordance with the practical task under consideration. DBIM serves as a nonlinear iterative reference for single-frame reconstruction. By repeatedly updating the background and re-solving the forward problem, it provides a more accurate approximation to complex scattering and, therefore, usually yields cleaner full-lesion reconstructions.

SR-TSVD addresses a different objective. Rather than maximizing the contour sharpness of an isolated frame, it is intended to maintain a stable evolving estimate over time under a constrained computational budget. The relevant trade-off is, therefore, not merely one of sharpness versus speed, but of single-frame reconstruction accuracy versus dynamic tracking capability. Under this interpretation, DBIM remains valuable as a gate-refresh reference, whereas SR-TSVD is more suitable for the continuous monitoring task addressed in this work. At the same time, SR-TSVD is not equivalent to a fixed-kernel linear method. Its gate-refresh mechanism can be viewed as an event-driven relinearization strategy because the background state and linearized operator are rebuilt when the residual remains elevated. This gives the method stronger adaptability to moderate mismatch than schemes with a fixed imaging kernel. As a result, SR-TSVD is more robust to weak nonlinearity and gradual evolution. When the mismatch remains moderate and the signal-to-noise ratio is adequate, repeated gate refreshes can help incorporate accumulated mismatch into the updated gate state, thereby supporting more stable monitoring.

This distinction is reflected directly in the computational profile. SR-TSVD alternates between millisecond-level non-refresh updates and second-level gate-refresh frames, whereas DBIM requires repeated re-linearization and multiple forward solves for each evaluated frame. In the numerical study, gate refreshes typically occurred every two to three frames, indicating that the gate state remained valid for a short but useful interval under gradual lesion evolution. Although a gate-refresh frame is much more expensive than a standard TSVD update, it is still considerably cheaper than applying DBIM to every monitored frame. The same tendency is preserved in hardware. Even though the eight-antenna prototype reduces the matrix size and lowers the reconstruction time relative to the 16-antenna simulation setting, DBIM still requires more than eight times the computation of a refreshed SR-TSVD update for a single reconstructed frame. Accordingly, SR-TSVD is better suited to real-time tracking, whereas DBIM is more appropriate for applications in which higher single-frame accuracy is prioritized over computational speed.

The refresh threshold directly controls the trade-off between reconstruction stability and computational cost. In the present work, $\eta_{U}$ and $C_{\mathrm{th}}$ were selected empirically so that the gate state could remain valid for gradually evolving targets without causing unnecessary gate refreshes. These parameter values were chosen from coarse parameter sweeps under the tested scenarios. Because the residual may be influenced by both lesion evolution and measurement noise or modeling mismatch, the refresh mechanism is intended to preserve the validity of the gate-linearized model during monitoring. For lesion evolution with different rates, the gate parameters would need to be adjusted accordingly. If the threshold is too low, the method may over-refresh and lose part of its computational advantage. If it is too high, model mismatch may accumulate, and the first-order gate-linearized approximation may become unreliable before the gate state is updated. The current results indicate that the chosen thresholds provide a useful balance for the tested scenarios, but they still should not be viewed as universal. Their optimal values depend on the measurement cadence, the expected lesion evolution rate, and the level of hardware variability. An adaptive threshold strategy based on online residual statistics would, therefore, be a meaningful direction for future work.

The present study also clarifies the roles of sparse-array design and narrow-band frequency selection. The hardware validation uses an eight-element array, whereas the main numerical study uses 16 elements. This reduction does not correspond to a simple linear degradation, nor does it imply that conclusions obtained from 16 elements can be transferred unchanged to eight elements. The additional numerical study on antenna count shows that the effect of array size is strongly nonlinear. The gain from moving out of the very sparse regime is much more pronounced than the gain obtained once sampling has already become moderately dense. Accordingly, the main quality gap discussed here refers specifically to the gap between the 16-element numerical reconstructions and the eight-element phantom results. Its dominant source is the reduced angular sampling of the sparser hardware aperture, compounded by measurement nonidealities. This gap is, therefore, not merely a generic discrepancy between simulation and experiment, but a concrete limitation of sparse-array monitoring.

A similar interpretation applies to the frequency setting. The present study uses a narrow three-frequency set around 1 GHz. This choice is consistent with the narrow-band approximation adopted in the model and helps reduce sensitivity to dispersion over the monitored band. The additional frequency diversity study shows that, for the sparse eight-antenna case, introducing a small number of nearby frequencies improves reconstruction relative to the single-frequency case, whereas adding more frequencies yields only limited further benefit at a noticeably higher computational cost. A wider bandwidth could improve resolution and conditioning in principle, but it would also require explicit modeling of frequency-dependent contrast and dispersion and would further increase the cost of gate-refresh frames. The present frequency setting is chosen as a monitoring-oriented compromise that balances reconstruction benefit, dispersion control, and computational cost.

Several limitations remain and help define the scope of the present conclusions.

(1) Both the numerical model and the image reconstruction are two-dimensional. Although the proposed SR-TSVD framework can, in principle, be extended to three dimensions, the present study adopts a two-dimensional setting for controlled mechanism validation and for consistency with the current phantom setup. This setting does not capture out-of-plane scattering, three-dimensional skull heterogeneity, or the full anatomical complexity of the head. Its implication for SR-TSVD is not only a possible loss of image fidelity. In a true 3-D setting, gate-refresh frames would require substantially larger forward solves and system matrices, so the current cost asymmetry between non-refresh updates and gate refreshes could become more restrictive. At the same time, stronger background heterogeneity may reduce the validity range of the gate-linearized distorted Born approximation, especially near highly contrasting interfaces.
(2) SR-TSVD relies on the assumption of weak incremental scattering within each gate cycle. This assumption is reasonable when the lesion evolves gradually and when the gate is refreshed before the residual grows excessively. Its validity becomes weaker when abrupt changes occur, when multiple strong scatterers evolve simultaneously, or when a small concurrent change is masked by a larger dominant variation. In such cases, the residual may grow faster than the dwell counter can respond, and the method may enter a regime in which the current gate state no longer provides an adequate linearization background. The practical signature of this behavior is sustained residual growth, together with degraded localization or missed weak increments before refresh. The present study, therefore, establishes only a qualitative operating boundary for admissible lesion evolution under the tested settings, rather than a quantitative upper bound. Deriving such a bound would require a more detailed analysis of contrast magnitude, sampling density, and refresh cadence. For scenarios involving more rapid target evolution or more irregular lesion geometry, possible improvements may include more frequent gate refreshes or the introduction of a small number of iterative corrections within each gate cycle to better accommodate nonlinear scattering effects.
(3) The phantom experiment remains a simplified validation. Although it is suitable for testing dynamic trends and hardware repeatability, it does not reproduce several interfaces and motions that are important in vivo. The omitted interfaces include the skull–brain boundary, the CSF layer, and the contrast between gray and white matter. The most concerning forms of motion include bulk patient motion and small repeated changes in coupling geometry, both of which can alter the residual independently of lesion evolution and may trigger unnecessary gate refreshes or induce structured artifacts. These factors indicate the need for validation on more realistic models in the next stage of this research.
(4) The present framework still leaves room for further improvement. Its current strength lies in combining a physics-based gate state with lightweight state-referenced updates, which makes repeated monitoring feasible under sparse sampling. More broadly, SR-TSVD can be viewed as a model-based dynamic inverse strategy that updates an internal reference state rather than reconstructing every frame independently from scratch. A promising next step is, therefore, to strengthen the present framework with better temporal constraints, adaptive gate-refresh policies, or interpolation-assisted sparse-array strategies so that stability, speed, and spatial detail can be improved jointly.

In summary, SR-TSVD makes dynamic microwave monitoring feasible under sparse multistatic sampling by separating the reconstruction process into frequent lightweight non-refresh updates and infrequent gate-refresh steps. Within this framework, state referencing improves temporal consistency, whitening enhances robustness to channel imbalance, and residual-driven gate refresh helps preserve the validity of the linearized model. These features together establish SR-TSVD as an effective framework for stable dynamic tracking under realistic computational constraints.

## Figures and Tables

**Table 1:** Dynamic-tracking quantitative comparison in numerical simulations.

Method	MSE, Mean ± std	SSIM, Mean ± std
S-TSVD	0.028 ± 0.006	0.112 ± 0.031
FD-WTSVD	0.033 ± 0.004	0.109 ± 0.045
SR-TSVD	0.012 ± 0.003	0.188 ± 0.033

**Table 2:** Full-lesion quantitative comparison on refresh frames in numerical simulations.

Method	MSE, Mean ± std	SSIM, Mean ± std
SR-TSVD	0.021 ± 0.004	0.147 ± 0.031
DBIM	0.011 ± 0.002	0.194 ± 0.045

**Table 3:** Average reconstruction time per frame in numerical simulations.

Method	Non-Refresh Frame Time (ms), Mean ± std	Refresh Frame Time (s), Mean ± std
S-TSVD	3.194 ± 0.041	28.854 ± 0.843
FD-WTSVD	3.188 ± 0.038	-
SR-TSVD	3.203 ± 0.042	28.910 ± 0.796
DBIM	-	241.864 ± 18.278

Note: Non-refresh frames refer to frames without gate refresh, and refresh frames refer to frames where the gate is updated and system matrices are rebuilt. DBIM is reported as the reconstruction time for full-lesion contrast imaging on the evaluated frames.

**Table 4:** Quantitative comparison for different antenna counts in the single-lesion growth sequence.

Antenna Count	MSE, Mean ± std	SSIM, Mean ± std
4	0.2843 ± 0.064	0.027 ± 0.019
6	0.1345 ± 0.031	0.042 ± 0.032
8	0.0392 ± 0.006	0.083 ± 0.025
12	0.0196 ± 0.002	0.141 ± 0.022
16	0.0111 ± 0.002	0.191 ± 0.024
24	0.0108 ± 0.002	0.204 ± 0.025

**Table 5:** Comparison between single-frequency and multi-frequency inversion.

Antenna Count	Frequency Setting (GHz)	MSE, Mean ± std	SSIM, Mean ± std	Refresh Frame Runtime (s), Mean ± std
8	1.00	0.0459 ± 0.009	0.056 ± 0.029	8.475 ± 0.241
8	0.95/1.00/1.05	0.0392 ± 0.006	0.083 ± 0.025	23.166 ± 0.683
8	0.95/0.975/1.00/1.025/1.05	0.0374 ± 0.006	0.079 ± 0.023	40.067 ± 1.022
16	1.00	0.0118 ± 0.002	0.186 ± 0.024	9.321 ± 0.249
16	0.95/1.00/1.05	0.0111 ± 0.002	0.191 ± 0.024	27.375 ± 0.733
16	0.95/0.975/1.00/1.025/1.05	0.0106 ± 0.001	0.204 ± 0.025	44.168 ± 1.175

**Table 6:** Inclusion geometry in the hardware sequence.

Center (cm)	Quantity	*t* = 1	*t* = 2	*t* = 3	*t* = 4	*t* = 5	*t* = 6	*t* = 7	*t* = 8
(2, −4)	Volume (mL)	10	20	30	20	10	/	/	/
	Diameter (cm)	2.67	3.37	3.86	3.37	2.67	/	/	/
(−1.5, 5)	Volume (mL)	/	/	10	20	30	30	20	10
	Diameter (cm)	/	/	2.67	3.37	3.86	3.86	3.37	2.67

Note: A slash indicates absence of the inclusion at that frame.

**Table 7:** Dynamic-tracking quantitative comparison in hardware experiments.

Method	MSE, Mean ± std	SSIM, Mean ± std
S-TSVD	0.098 ± 0.021	0.033 ± 0.012
FD-WTSVD	0.147 ± 0.025	0.038 ± 0.018
SR-TSVD	0.042 ± 0.012	0.068 ± 0.014

**Table 8:** Full-lesion quantitative comparison on refresh frames in hardware experiments.

Method	MSE, Mean ± std	SSIM, Mean ± std
SR-TSVD	0.053 ± 0.018	0.056 ± 0.015
DBIM	0.028 ± 0.007	0.077 ± 0.011

**Table 9:** Reconstruction cost in hardware experiments.

Method	Non-Refresh Frame Time (ms), Mean ± std	Refresh Frame Time (s), Mean ± std
S-TSVD	2.018 ± 0.027	23.247 ± 0.624
FD-WTSVD	2.114 ± 0.027	-
SR-TSVD	2.121 ± 0.031	23.250 ± 0.622
DBIM	-	189.681 ± 14.182

## Data Availability

The data are available upon reasonable request from the corresponding author.
